# Patient-Controlled Analgesia in ICU: A Scoping Review

**DOI:** 10.3390/jpm16020109

**Published:** 2026-02-12

**Authors:** Angela Califano, Riccardo Caldonazzo, Miriam Gotti, Giovanni Sabbatini, Andrea Galimberti, Pezzi Angelo, Paolo Formenti

**Affiliations:** 1 School of Medicine and Surgery, University of Milano-Bicocca, 20126 Milano, Italy; 2 SC Anestesia, Rianimazione e Terapia Intensiva, ASST Nord Milano, Ospedale Bassini, 20097 Cinisello Balsamo, Italy

**Keywords:** patient-controlled analgesia, Intensive Care Unit, personalized pain management

## Abstract

**Background/Objectives**: Patient-Controlled Analgesia (PCA) is a well-established strategy for managing postoperative pain, but its use in the Intensive Care Unit (ICU) remains poorly defined, poorly standardized, and fragmented. The aim of this scoping review is to map the extent, nature, and characteristics of the available evidence on the use of PCA in ICU patients, identifying key areas of uncertainty and knowledge gaps that require further study. **Methods**: Scoping review reported according to the PRISMA-ScR guidelines. **Results**: 12 relevant studies were identified. Available evidence suggests that PCA can provide pain control comparable to traditional techniques in post-cardiac surgery patients in the ICU, while data on its use in non-surgical patients are limited. The studies reported good feasibility and a generally favorable safety profile, with a low incidence of significant respiratory events thanks to intensive monitoring. Methodological variability prevents direct comparisons between studies. **Conclusions**: PCA supports personalized pain management based on patient-specific clinical conditions and response. However, more standardized studies are needed to define its role.

## 1. Introduction

Patient-controlled analgesia (PCA) is a well-established modality for the management of acute pain and is now used in numerous clinical settings [1]. PCA allows the patient to self-administer predetermined doses of analgesic via a button connected to a programmable pump [2]. Although initially introduced for the postoperative intravenous administration of morphine, over time its use has extended to the subcutaneous route [3,4] to central and peripheral locoregional techniques [5] and, more recently, to some chronic pain conditions [6]. The main advantage of PCA is the possibility of ensuring faster and more personalized pain control compared to intermittent administrations performed by healthcare professionals, reducing fluctuations in plasma opioid levels and periods of inadequate analgesia [7].

Despite these potential benefits, the literature specifically dedicated to the use of PCA in Intensive Care remains limited. This is partly due to the need for patient cooperation in the postoperative period, a requirement not always compatible with the conditions of critically ill patients, who may be intubated, undergoing assisted ventilation or undergoing extubation [8]. The application of PCA in ICU also presents several critical issues. Critically ill patients show heterogeneous responses to pain and analgesic drugs and require a delicate balance between pain control, sedation and respiratory safety [9]. The effectiveness of PCA presupposes that the patient is alert, cooperative and able to understand and correctly use the device, a requirement that can be compromised by clinical conditions, sedative drugs or altered mental status [10]. Recent perioperative ICU studies in cardiac surgery continue to report heterogeneous results, confirming that robust ICU-specific evidence remains limited [11,12].

PCA also requires careful monitoring to prevent dosing errors and ensure appropriate use, potentially increasing the workload of the staff [13]. It is not suitable for deeply sedated or intubated patients, who are unable to independently manage pain administration. Further risks are related to possible device malfunctions or programming errors [14]. Finally, the safe implementation of PCA in the ICU requires structured training programs for patients and healthcare professionals. Adequate education on the device’s use and continuous monitoring is essential to minimize adverse events and maximize clinical benefits [15].

Given the heterogeneity of ICU patients, PCA may represent an individualized approach to pain management, allowing adjustment according to clinical conditions and response. This scoping review maps the available evidence on the use of PCA in the ICU, analyzing its effects on pain control, clinical outcomes, and safety, and identifies priority areas for future research.

## 2. PCA: The Basis

### 2.1. Principles of PCA Functioning

PCA is based on programmable pumps that allow patients to self-administer small doses of analgesics (most often intravenous opioids) within predefined safety limits [1]. Compared with intermittent nurse-controlled dosing, PCA maintains more stable plasma concentrations and allows a more individualized analgesic profile while limiting adverse effects. PCA can be delivered through different routes, including intravenous or central venous access, epidural catheters (PCEA), perineural catheters (PCCA), transdermal systems, or sublingual devices. While intravenous PCA remains the most common modality, the introduction of PCEA and PCCA has expanded its use in postoperative and trauma settings [16,17,18], whereas transdermal systems may be useful in scenarios requiring prolonged non-invasive analgesia.

**Figure 1:**
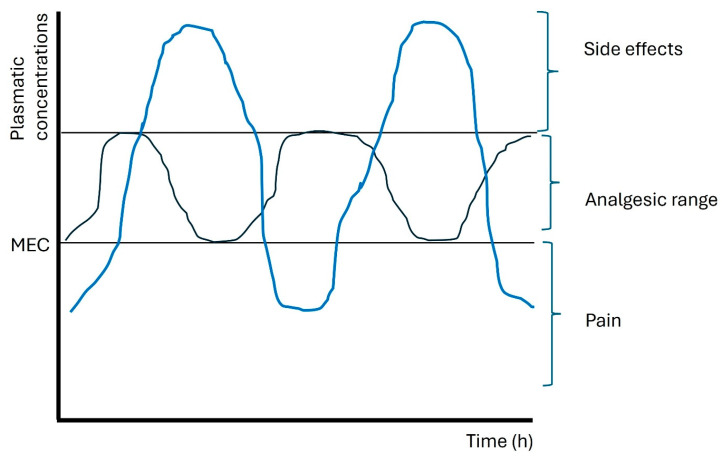
Plasma opioid concentration trends over time with different administration methods.

Regardless of the device, PCA systems share key elements such as a reservoir, a programmable interface, a safety lockout mechanism, and an activation button [19]. Pump programming typically includes a loading dose to reach the minimum effective concentration (MEC), followed by patient-triggered bolus doses (reliever doses) with lockout intervals to prevent overdose [18]. Some systems also incorporate a continuous basal infusion to ensure background analgesia, along with maximum hourly limits that provide additional safety and prompt reassessment if they are reached (Figure 1) [20,21].

The most frequently used drugs in PCA are μ-opioid receptor agonists such as morphine, fentanyl, hydromorphone, and related agents [22,23]. Adjuvants (including ketamine, naloxone, clonidine, magnesium, ketorolac, lidocaine, or droperidol) may be added to enhance analgesia or reduce opioid-related adverse effects [24]. Local anesthetics, such as bupivacaine, levobupivacaine, and ropivacaine, are mainly employed in epidural and perineural PCA modalities [25].

### 2.2. Patient Selection and Stratification in a Personalized Medicine Framework

Appropriate patient selection is central to personalized analgesia in the ICU, allowing stratification according to cognitive status, respiratory risk, comorbidities, and individual susceptibility to opioid-related adverse effects. Before initiating PCA, the patient must be able to understand its use and to activate the demand button independently.

PCA is therefore unsuitable in the presence of delirium, dementia, or relevant cognitive impairment [26]. Uncontrolled psychiatric conditions and strong refusal to self-administer opioids may also represent contraindications. Several factors increase the risk of respiratory depression during PCA, including advanced age, obesity, untreated obstructive sleep apnea, smoking, reduced functional independence, and cardiopulmonary disease [27]. Additional risk arises from renal or hepatic impairment, which alters opioid metabolism, as well as chronic respiratory conditions such as severe COPD or uncontrolled asthma [28]. Extrinsic factors may further contribute, including rapid administration of high doses in opioid-naïve patients or the addition of high basal infusions in those already receiving chronic opioids.

The first 24 postoperative hours represent the period of greatest vulnerability due to residual anesthetic effects. Long procedures (>2 h) and surgeries limiting deep ventilation may further increase respiratory complications. In opioid-naïve patients, basal infusions combined with PCA may increase respiratory depression without improving analgesia [29]. In the ICU, however, continuous monitoring mitigates some risks and allows more individualized titration. Structured institutional protocols and checklists facilitate systematic risk assessment, and personnel must be trained in PCA monitoring and troubleshooting, avoiding overreliance on technology [30]. Education of patients and caregivers is also essential, particularly regarding the risks of “auxiliary activation,” i.e., unauthorized activation of the button. Adequate training combined with continuous monitoring remains key to ensuring safety and effectiveness of PCA in the critical care setting.

The figure illustrates the plasma concentration trends of an opioid over time in relation to the minimum effective threshold (MEC), the optimal analgesic range, and the level beyond which adverse effects increase. The blue curve represents intermittent administration, which causes marked peaks and troughs in concentrations, with the risk of both periods of analgesic ineffectiveness and exposure to levels associated with side effects. The black curve, however, shows a more stable profile, with concentrations maintained most of the time within the analgesic range, reducing fluctuations and minimizing both episodes of uncontrolled pain and potential adverse effects.

## 3. Materials and Methods

This scoping review was conducted following the PRISMA-ScR recommendations, with the aim of summarizing the existing literature on the use of PCA in critically ill adult patients. Although registration is not mandatory for scoping reviews, the protocol was nonetheless documented prospectively in the PROSPERO database (CRD42024562554). The completed checklist is provided as Supplementary File (Table S1).

We included randomized controlled trials, quasi-experimental studies and observational studies evaluating patient-controlled analgesia (PCA) in adult ICU patients. Studies enrolling postoperative or medical ICU patients were eligible, provided that systemic PCA was used for analgesia. Comparators included nurse-controlled or continuous opioid administration, alternative PCA regimens, or no comparator. We excluded pediatric populations, non-ICU settings (e.g., post-anesthesia care unit without subsequent ICU stay), case reports, editorials, animal studies and articles not reporting analgesic or opioid-related outcomes. Only full-text articles published in English were considered.

The literature search was performed in PubMed/MEDLINE, Embase, Scopus and the Cochrane Library, until 30 May 2025. Reference lists of included articles and relevant reviews were hand-searched to identify additional eligible studies. Search terms included “patient-controlled analgesia”, “PCA”, “critical care” and “ICU”. The full electronic search strategy for the main database is provided in the Supplementary Materials (Table S2). No automation tools or gray literature databases were used. Two reviewers (A.C. and P.F.) independently screened titles, abstracts and full texts according to the eligibility criteria. Disagreements were resolved by discussion.

Data were extracted into a standardized form including study characteristics (author, year, country, setting, study design), population features (ICU type and surgical procedure), PCA modality (drug, dosing, infusion settings), comparator, and outcomes (pain scores, opioid consumption, complications, delirium, ICU length of stay, patient satisfaction, feasibility and adverse events). When available, information on sample size, analgesic agents and timing of assessment was also collected. The primary outcomes were pain intensity and opioid consumption. Secondary outcomes included ICU complications (e.g., respiratory depression, postoperative delirium), ICU length of stay, patient satisfaction, feasibility and adverse events.

## 4. Results

**Figure 2:**
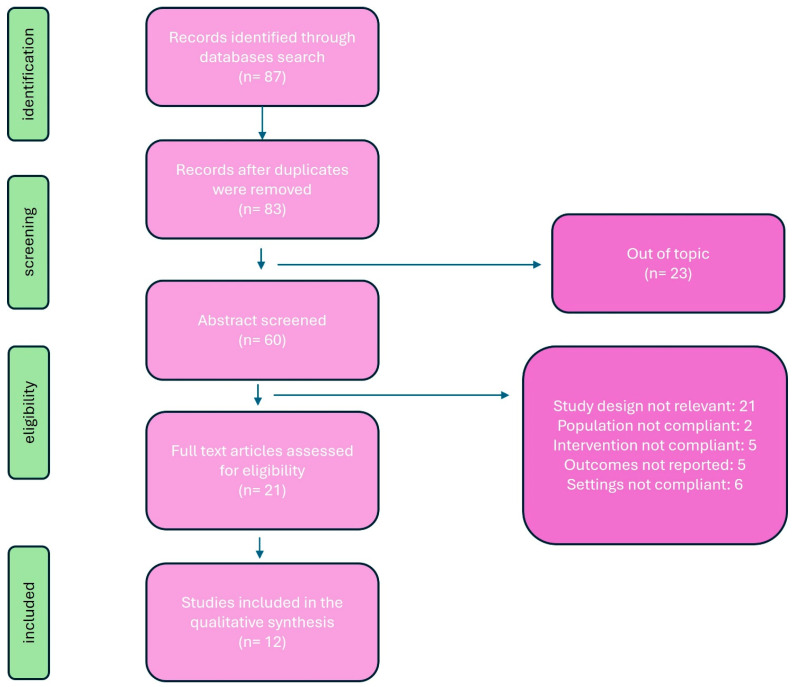
PRISMA-ScR flowchart of the study selection process.

The initial search identified 87 records, of which 83 remained after removal of duplicates. After title and abstract screening, 23 studies were excluded. Sixty full-text articles were assessed for eligibility; 48 were excluded for wrong study design (*n* = 21), wrong population (*n* = 2), wrong intervention (*n* = 5), no relevant outcomes (*n* = 5), or non-ICU setting (*n* = 6). Twelve studies met the inclusion criteria and were included in the qualitative synthesis. The study selection process is summarized in Figure 2. The 12 included studies were published between 1994 and 2023 and comprised randomized controlled trials, quasi-experimental studies, observational cohorts, and one systematic review. Most studies evaluated postoperative populations, predominantly after cardiac surgery, where PCA was compared with nurse-controlled opioid administration. PCA regimens varied and included intravenous morphine, hydromorphone, and sufentanil, with or without adjuncts such as ketamine, naloxone, or dexmedetomidine. Only one study evaluated PCA in non-surgical ICU patients. Sample sizes were generally small, and clinical heterogeneity was substantial, particularly regarding PCA settings, comparator strategies, and outcome reporting. The main characteristics of the included studies are presented in Table 1.

Given these limitations, a quantitative synthesis would not have been appropriate or methodologically sound. For this reason, the analysis was conducted as a scoping review, with an exclusively qualitative synthesis of the available evidence.

The diagram illustrates the steps in the process of identifying, screening, and including studies according to the PRISMA-ScR guidelines. The database search yielded 87 records, reduced to 83 after removing duplicates. Sixty abstracts were evaluated, of which 23 were excluded due to irrelevance. Twenty-one articles were reviewed in full: of these, nine were excluded due to irrelevant study design, two for non-compliant population, five for inappropriate intervention, five for lack of outcomes, and six for non-compliant settings. In total, 12 studies met the eligibility criteria and were included in the qualitative synthesis.

## 5. Discussion

### 5.1. Efficacy of Pain Relief

Most studies evaluating the efficacy of PCA have been conducted in the postoperative setting outside of the Intensive Care Unit. A recent systematic review has highlighted, with variable quality of evidence, that PCA represents an effective alternative to non-patient-controlled systemic analgesia for the management of postoperative pain [7].

The few studies identified specifically for the ICU largely come from the cardiac surgery context, where PCA is more easily applicable thanks to the greater clinical stability of patients in the postoperative period and the presence of a higher nurse-patient ratio compared to ordinary wards. These factors ensure continuous monitoring and timely administration of analgesia, conditions that favor the study of PCA in the period immediately following surgery. From a practical standpoint, postoperative ICU patients (particularly after cardiac surgery) appear to be the most suitable candidates for PCA, as they are more likely to be awake, cooperative, and clinically stable. In contrast, evidence supporting PCA use in non-surgical ICU patients remains scarce and limited to highly selected cases.

Several studies have compared PCA with nurse-controlled analgesia. A randomized trial also evaluated a patient-controlled sublingual sufentanil system versus nurse-controlled morphine infusion up to ICU discharge, showing adequate analgesia with lower opioid exposure [12]. In a trial of 80 patients undergoing cardiac surgery, no significant differences were observed in pain scores at rest or in movement, adverse effects, daily morphine requirements, or analgesic efficacy during chest physiotherapy [41]. Similarly, in a randomized trial of 72 patients undergoing elective cardiac surgery, PCA showed comparable benefits to nurse-controlled morphine titration [31]. A further RCT of 66 patients found no difference between PCA and nurse-controlled analgesia using continuous morphine infusion [32]. Pettersson et al. [33] also compared PCA with nurse-administered intravenous analgesia after cardiac surgery, observing improved pain control in patients treated with PCA, a result maintained even after transfer to the ward. In contrast, Tsang et al. [39] found no significant differences between PCA and nurse-administered intravenous morphine for pain control, lung function, or opioid requirements.

A more recent meta-analysis of over 600 patients showed a reduction in pain scores at 48 h in patients treated with PCA compared to nurse-administered analgesia, with no differences in terms of duration of ventilation, ICU or hospital stay, satisfaction, sedation, nausea, vomiting, respiratory depression, severe pain, treatment discontinuations, or mortality [35]. However, many of these outcomes were not fully reported in the primary studies.

Outside the postoperative setting, only a few studies have explored the use of PCA in alert and spontaneously breathing critically ill patients. In a cohort of 281 patients, PCA with morphine was started in 100 cases (35%); despite some interruptions due to ineffectiveness or incomplete evaluations, a significant reduction in pain scores was observed after titration, with stable vital signs throughout the study [36].

### 5.2. Opioid Consumption Effects

Despite one of the critical benefits of PCA is its potential to reduce overall opioid consumption, this effect has not been extensively studied as a primary outcome in studies involving ICU patients. Some of these studies have predominantly evaluated the addition of drug combinations to assess their effect on lower opioid doses. For instance, in a randomized, double-blinded study in a surgical ICU, the authors measured 4 h cumulative morphine doses and VAS scores comparing patients received either morphine PCA with placebo or with ketamine [37]. In a previously RCT cited paper involving 72 patients undergoing elective cardiac surgery, morphine consumption was measured at 0–24 h and 24–48 h post-surgery [31]. The trial revealed no significant difference in morphine consumption between PCA and nurse-titrated infusion.

In a study comparing PCA with or without a background infusion of morphine after cardiac anesthesia, postoperative morphine consumption at more than 40 h, was significantly lower in the PCA alone group (29.43 ± 12.57 mg) compared to the PCA plus continuous infusion group (50.14 ± 16.44 mg) [42]. Similarly, in the study by O’Halloran [32], morphine consumption was measured at 24 h, showing that PCA group also consumed significantly more morphine. In the metanalysis which compared PCA with conventional nurse-controlled analgesia, the cumulative morphine equivalents consumed significantly increased with PCA at both 24 and 48 h [35].

Finally, in a study on patients undergoing lumbar discectomy, adding ultra-low-dose naloxone (0.25 μg/kg/h) to morphine PCA monitored in the post anesthesia care unit and subsequently in the ward, showed a significantly reduced morphine consumption compared to placebo [38]. Overall, the available studies report heterogeneous effects of PCA on total opioid consumption, with findings ranging from reduced to unchanged or even increased opioid use, largely depending on study design, comparator strategies, and dosing protocols. In non-intubated trauma ICU patients, ketamine-based PCA significantly reduced cumulative opioid requirements compared with hydromorphone PCA, at the expense of more hallucinations [43].

### 5.3. Adverse Side Effects

A recent meta-analysis in the general population compared different opioid by evaluating nausea, vomiting, pruritus, sedation and respiratory depression [44]. Overall, opioids showed minimal differences compared to morphine, with a few exceptions (e.g., buprenorphine with greater nausea/vomiting and fentanyl with lower risk). Respiratory depression was rare, and the most relevant differences concerned satisfaction and pruritus profiles. It is important to remember that these results refer to the general population and not specifically to patients in the ICU.

Another recent meta-analysis of ICU patients after cardiac surgery found no significant differences between groups in terms of duration of ventilation, ICU and hospital stay, sedation, satisfaction, or incidence of postoperative adverse effects [35], although results were reported inconsistently across studies. Some reports indicate a lower incidence of nausea with morphine-based PCA compared with nurse-administered administration [31,32], and the addition of a basal infusion does not appear to increase common side effects [42].

Studies evaluating different medications in PCA in ICU settings report variable tolerability profiles: in spinal neurosurgery patients, as-needed ketamine-based PCA was associated with fewer adverse effects than mixed opioid-ketamine PCA [44]; in thoracic surgery patients, hydromorphone PCA showed a lower incidence of pulmonary complications, particularly pneumonia, compared to sufentanil PCA, with no differences in nausea or vomiting [39]. The addition of low-dose naloxone to PCA has been proposed to reduce nausea and vomiting [38], although other studies have not confirmed a clear benefit. Overall, the incidence of respiratory events was low, likely due to the intensive monitoring provided by the ICU environment.

In non-ICU settings, meta-analyses could not evaluate respiratory depression because most studies reported zero events [45]. Furthermore, heterogeneity in the definition of respiratory effects must be considered, ranging from simple respiratory rate thresholds to complications such as atelectasis, lung infections or respiratory failure [39]. In a large study of patients undergoing coronary artery bypass grafting, radiographic atelectasis scores were significantly better in the PCA-treated groups compared to controls [46].

### 5.4. Secondary Outcomes

Some studies suggest that improved pain management with PCA may promote faster recovery and, in some cases, reduced ICU stay [47]. Comparisons of transdermal fentanyl systems with intravenous PCA with morphine, for example, have shown potential benefits on recovery times after abdominal or pelvic surgery.

However, several other studies have found no significant differences in ICU or hospital length of stay between PCA and traditional analgesia [7,31,48,49] nor in early discharge rates or 30-day mortality [40]. Recent randomized data in cardiac surgery patients, where PCIA was part of postoperative management, reported differences in ICU length of stay and cognitive outcomes across analgesic strategies, reinforcing the need for standardized ICU-focused protocols [50].

Regarding delirium, available evidence does not indicate a specific impact of PCA on its incidence [51]. The pathophysiology of delirium remains complex and not fully understood; the most accredited hypothesis concerns an alteration of central neurotransmission, with a reduction in cholinergic activity and dopaminergic hyperactivity, with the possible involvement of other mediators such as serotonin and GABA [52]. It is also known that uncontrolled pain represents a risk factor for the development of delirium [53]. Only one recent study directly evaluated the effect of PCA on delirium using an infusion of dexmedetomidine [40]: in 236 patients undergoing thoracoabdominal surgery, perioperative administration of the drug significantly reduced the incidence of delirium compared to the control group. Delirium outcomes have also been explored in other high-risk cardiac procedures using different analgesic strategies [54].

## 6. Practical Recommendations

**Figure 3:**
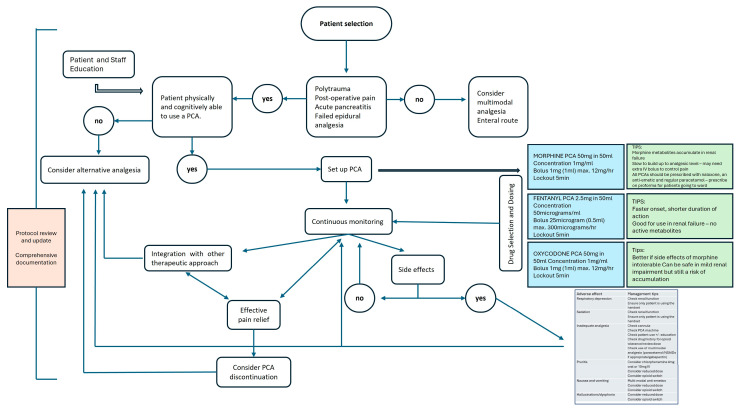
Practical algorithm for the management of Patient-Controlled Analgesia in the ICU.

The definition of an operational protocol for the use of PCA in Intensive Care requires a structured approach. We have tried to provide input for the reader and to summarize a practical flow chart (Figure 3). The first step consists of patient selection, carefully evaluating the physical and cognitive ability to use the device independently; patients with altered mental status or cognitive deficits are not suitable candidates [13]. Once eligible patients have been identified, staff and patient training plays a central role, including instructions on PCA operation, compliance with dosage limits and recognition of adverse effects [55]. The flow chart also highlights the importance of drug choice and correct setting of infusion parameters, including loading dose, lockout interval and maximum hourly limits, essential aspects to ensure effective and safe analgesia [56]. After initiating PCA, continuous monitoring is necessary, with frequent assessment of sedation, respiratory rate, nausea, vomiting and other potential side effects, supported by dedicated monitoring systems. The timely management of adverse effects, also represented in the diagram, requires clear and shared protocols that define interventions for nausea, pruritus, or insufficient pain control [57]. The flow chart also includes the possibility of integrating PCA with additional therapeutic strategies, both pharmacological and non-pharmacological, to optimize pain control and reduce opioid requirements.

Finally, protocol management requires periodic review and accurate documentation of clinical progress and patient responses, which are essential for ensuring continuity of care, improving safety, and adopting updates based on the latest evidence. Overall, the proposed algorithm represents a practical translation of personalized medicine principles into daily ICU practice, combining patient stratification, individualized risk assessment, and tailored analgesic strategies within a structured decision-making process. The future role of PCA in the ICU may be influenced by advanced monitoring approaches, including algorithm-based and artificial intelligence–supported systems, which have been proposed to improve early detection of opioid-induced respiratory depression [58]. Such technologies, combined with multimodal analgesia strategies, may further enhance the safety and feasibility of PCA in selected critically ill patients

The flow chart illustrates the decision-making process for the use of PCA in patients admitted to the ICU, including patient selection, assessment of cognitive and physical capacity for use of the device, staff and patient education, drug selection and infusion parameters, continuous monitoring, management of adverse effects, and integration with other therapeutic approaches. The diagram also includes guidelines for periodic protocol review and complete clinical documentation to ensure safety, analgesic efficacy, and continuity of care.

## 7. Conclusions

PCA appears to offer several advantages for patients admitted to the ICU, particularly regarding faster, more stable, and more satisfactory pain control perceived by eligible patients. Its impact on total opioid consumption remains variable across studies, although PCA may improve the timing and individualization of analgesic delivery. However, it should be emphasized that the current evidence base supporting PCA use in the ICU is largely derived from postoperative surgical populations.

Despite these positive aspects, the use of PCA in the ICU has been investigated with widely varying study designs, heterogeneous populations, and often incompletely reported outcomes. This variability makes it difficult to draw solid conclusions and highlights the need for greater protocol standardization.

Larger, more consistent studies are therefore needed to clarify the role of PCA, especially in medical ICU, define which patients may benefit most from it, and evaluate whether its use can contribute to broader improvements in the recovery process during the ICU stay.

## Figures and Tables

**Table 1:** Characteristics of the included studies assessing Patient-Controlled Analgesia in ICU.

Study	Setting & Population	Design	PCA Modality	Comparator	Main Outcomes Reported	Key Findings
Dawson et al., 1995 [20]	Post-abdominal surgery patients in ICU; adults requiring postoperative analgesia	RCT	IV PCA + continuous infusion	PCA alone/nurse-controlled	Pain scores; morphine consumption; complications	Adding continuous infusion to PCA did not improve analgesia and increased opioid use; similar safety profile.
Myles et al., 1994 [31]	Post-cardiac surgery ICU patients	RCT	IV PCA	Nurse-controlled infusion	Pain control; morphine use; satisfaction; side effects	PCA provided similar analgesia to nurse-controlled infusion; no differences in opioid use or complications.
O’Halloran et al.,1997 [32]	Cardiac surgery ICU	RCT	IV PCA	Nurse-controlled continuous morphine	Pain scores; opioid consumption; adverse events	No significant difference between PCA and continuous infusion in pain or respiratory events; PCA group consumed slightly more morphine.
Pettersson et al., 2000 [33]	Coronary artery bypass graft postoperative ICU	RCT	IV PCA	Nurse-controlled analgesia	Pain at rest/movement; morphine use	PCA produced better pain control, maintained after ICU discharge.
Tsang et al., 1999 [34]	Post-cardiac surgery ICU	Quasi-experimental	IV PCA	Nurse-administered IV morphine	Pain scores; lung function; morphine consumption	No significant differences; PCA is considered feasible but not superior.
Bainbridge et al., 2006 [35]	Meta-analysis including ICU cardiac surgery subgroups	Meta-analysis	PCA (various opioids)	Nurse-controlled analgesia	Multiple (pain, opioid use, stay, adverse events)	PCA reduced pain to 48 h but did not change ICU/hospital stay, ventilation time, complications, or satisfaction.
Lefrant et al., 2000 [36]	Mixed ICU population; spontaneously breathing patients	Observational	IV PCA (morphine)	None	Pain scores; feasibility; adverse events	PCA is feasible in 35% of eligible ICU patients; significant pain reduction; vital signs remained stable.
Guillou et al., 2003 [37]	Post–major abdominal surgery ICU	RCT	PCA morphine + low-dose ketamine	PCA morphine alone	Morphine consumption; pain scores; side effects	Low-dose ketamine reduced morphine use without increasing adverse effects.
Firouzian et al., 2018 [38]	Post–lumbar discectomy ICU/PACU	Double-blind RCT	PCA morphine + ultra-low-dose naloxone	PCA morphine + placebo	Morphine use; pain; nausea/vomiting	Naloxone reduced opioid consumption and postoperative nausea.
Yan et al., 2018 [39]	Thoracic surgery ICU patients	Quasi-experimental	PCA with hydromorphone or sufentanil	Comparison between PCA regimens	Pulmonary complications; pain; adverse effects	Hydromorphone PCA was associated with fewer pulmonary complications, similar analgesia.
Xie et al., 2023 [40]	Elderly patients after thoracoabdominal surgery	RCT	PCA + dexmedetomidine infusion	PCA without dexmedetomidine	Delirium; pain; opioid effects	Dexmedetomidine PCA significantly reduced delirium incidence.

## Data Availability

No new data were created or analyzed in this study. All data supporting the findings of this scoping review are available within the cited literature.

## Supplementary Materials

The following supporting information can be downloaded at: https://www.mdpi.com/article/10.3390/jpm16020109/s1, Table S1: prospero checklist; Table S2: electronic search strategy for the main database.

## Abbreviations

The following abbreviations are used in this manuscript:

PCA	Patient-Controlled Analgesia
ICU	Intensive Care Unit
IV	Intravenous
PCEA	Patient-Controlled Epidural Analgesia
PCCA	Patient-Controlled Catheter Analgesia
RCT	Randomized Controlled Trial
MEC	Minimum Effective Concentration
RR	Respiratory Rate
VAS	Visual Analog Scale
LOS	Length of Stay
OSA	Obstructive Sleep Apnea
CAM-ICU	Confusion Assessment Method for the ICU
RASS	Richmond Agitation-Sedation Scale
PRISMA-ScR	Preferred Reporting Items for Systematic Reviews and Meta-Analyses Extension for Scoping Reviews
